# Phylogeographic Analyses Reveal the Early Expansion and Frequent Bidirectional Cross-Border Transmissions of Non-pandemic HIV-1 Subtype B Strains in Hispaniola

**DOI:** 10.3389/fmicb.2019.01340

**Published:** 2019-06-26

**Authors:** Gonzalo Bello, Ighor Arantes, Vincent Lacoste, Marlene Ouka, Jacques Boncy, Raymond Césaire, Bernard Liautaud, Mathieu Nacher, Georges Dos Santos

**Affiliations:** ^1^ Laboratório de AIDS e Imunologia Molecular, Instituto Oswaldo Cruz, FIOCRUZ, Rio de Janeiro, Brazil; ^2^ Laboratoire des Interactions Virus-Hôtes, Institut Pasteur de la Guyane, Cayenne, French Guiana; ^3^ Virology Laboratory, EA 4537, Martinique University Hospital, Fort de France, Martinique; ^4^ Laboratoire National de Santé Publique, Ministère de la Santé Publique et de la Population, Port-au-Prince, Haiti; ^5^ Training Unit, GHESKIO Centers, Port-au-Prince, Haiti; ^6^ Coordination Régionale de la lutte contre le VIH (COREVIH) and Centre d’Investigation Clinique—CIC INSERM 1424, Centre Hospitalier de Cayenne “Andrée Rosemon”, Cayenne, French Guiana

**Keywords:** HIV-1, subtype B, non-pandemic, origin, phylodynamics, Haiti, Dominican Republic

## Abstract

The human immunodeficiency virus-type 1 (HIV-1) subtype B has probably been circulating on the island of Hispaniola since the 1960s, but information about the early viral history on this Caribbean island is scarce. In this study, we reconstruct the dissemination dynamics of early divergent non-pandemic subtype B lineages (designated B_CAR_) on Hispaniola by analyzing a country-balanced dataset of HIV-1 B_CAR_ *pol* sequences from Haiti (*n* = 103) and the Dominican Republic (*n* = 123). Phylogenetic analyses supported that B_CAR_ strains from Haiti and the Dominican Republic were highly intermixed between each other, although the null hypothesis of completely random mixing was rejected. Bayesian phylogeographic analyses placed the ancestral B_CAR_ virus in Haiti and the Dominican Republic with the same posterior probability support. These analyses estimate frequent viral transmissions between Haiti and the Dominican Republic since the early 1970s onwards, and the presence of local B_CAR_ transmission networks in both countries before first AIDS cases was officially recognized. Demographic reconstructions point that the B_CAR_ epidemic in Hispaniola grew exponentially until the 1990s. These findings support that the HIV-1 epidemics in Haiti and the Dominican Republic have been connected by a recurrent bidirectional viral flux since the initial phase, which poses a great challenge in tracing the geographic origin of the B_CAR_ epidemic within Hispaniola using only genetic data. These data also reinforce the notion that prevention programs have successfully reduced the rate of new HIV-1 transmissions in Hispaniola since the end of the 1990s.

## Introduction

The island of Hispaniola, shared by Haiti and the Dominican Republic, included in 2016 around 217,000 people living with the human immunodeficiency virus-type 1 (HIV-1), the etiologic agent of the acquired immunodeficiency syndrome (AIDS) (UNAIDS, 2013, 2017). Initially, AIDS cases recognized in Haiti (Pape et al., 1983) and the Dominican Republic (Koenig et al., 1987) mostly involved men who had sex with men, but these countries now have generalized epidemics predominantly driven by heterosexual sex (Figueroa, 2008). Despite the high HIV prevalence rates reported for the general population in Haiti (2.1%: 1.9–2.3%) and the Dominican Republic (1.0%: 0.7–1.4%) in 2016, significant declines of the HIV incidence rates were observed in both countries over the last decade (UNAIDS, 2013, 2017).

The HIV-1 epidemic in Hispaniola is dominated by subtype B (Nadai et al., 2009; Myers et al., 2012; Lopez et al., 2015). Genetic evidence suggests that the subtype B epidemic in Hispaniola, as in many other Caribbean countries, is mainly driven by the transmission of multiple early divergent non-pandemic subtype B lineages (designated “B_CAR_”), although the worldwide disseminated “B_PANDEMIC_” lineage also circulates (Cabello et al., 2014, 2015; Divino et al., 2016; Bello et al., 2018). The early dissemination dynamics of the HIV-1 subtype B epidemics between Haiti and the Dominican Republic remain largely unknown. Previous evolutionary analyses pointed that subtype B probably entered in the Americas through Haiti around the mid-1960s and then moved to other countries (Gilbert et al., 2007; Junqueira et al., 2011; Worobey et al., 2016), but no or very few (*n* < 15) subtype B sequences from the Dominican Republic were included in those studies. Another study with a large number of HIV-1 sequences from the Dominican Republic has shown that subtype B virus circulates in this country since the early 1960s (Lopez et al., 2015), a result comparable to the estimated age of the Haitian epidemic. This study, however, may have traced the age of the Haitian subtype B ancestor rather than of the Dominican one, given that it assumed that all subtype B infections in the Dominican Republic resulted from a single introduction and that the hypothesis of multiple independent viral introductions from Haiti was not formally tested.

The objective of this work was to reconstruct the early spatiotemporal dynamics of dissemination and demographic history of non-pandemic HIV-1 B_CAR_ lineages on the island of Hispaniola and to estimate the current degree of geographic compartmentalization of the B_CAR_ epidemic between Haiti and the Dominican Republic.

## Materials and Methods

### HIV-1 Subtype B *pol* Haitian Sequences

A total of 127 new HIV-1 subtype B *pol* sequences covering the complete protease (PR) and the first part of the reverse transcriptase (RT) regions (nucleotides 2253 to 3275 of reference strain HXB2) were obtained from adult patients at Port-au-Prince, Haiti, who underwent HIV genotyping tests at the Virology Laboratory of the University Hospital of Martinique (Fort-de-France, Martinique) between 2009 and 2014. All patients provided written informed consent and samples were anonymized as recommended in the study protocol approved by the Comité National d’Ethique du Ministère de la Santé Publique et de la Population de Haiti (13/07/2009). Only one sequence per subject was selected and all sites associated with major antiretroviral drug resistance were removed.

### HIV-1 Subtyping and Lineage Assignment

The subtype initially assigned to new Haitian sequences by the REGA HIV subtyping tool v.2 (de Oliveira et al., 2005) was confirmed by performing a maximum likelihood (ML) phylogenetic analysis with HIV-1 group M subtype reference sequences (Supplementary Table S1). We also tested for recombination using the Recombination Detection Program (RDP) v4.9 (Martin et al., 2005) with the default settings. Only statistically significant (*p* < 0.05) events supported by at least two methods were considered. HIV-1 subtype B *pol* sequences from Haiti were then aligned with subtype B sequences representative of the B_PANDEMIC_ and the B_CAR_ clades (Supplementary Table S2) selected from a previous study (Cabello et al., 2014) and subjected to ML phylogenetic analysis for lineage classification. The ML trees were reconstructed with the PhyML program (Guindon et al., 2010) using an online web server (Guindon et al., 2005) under the best fit nucleotide substitution model selected with the SMS tool (Lefort et al., 2017), the SPR branch-swapping algorithm of heuristic tree search, and the approximate likelihood-ratio test (aLRT) (Anisimova and Gascuel, 2006) of reliability tree topology. The ML trees were visualized using the FigTree v1.4 program (Rambaut, 2009).

### Analysis of Population Subdivision by Country

The HIV-1 B_CAR_ *pol* sequences from Haiti identified here were aligned with B_CAR_ sequences from Haiti (*n* = 12) and the Dominican Republic (*n* = 123) previously characterized (Cabello et al., 2014). A Bayesian phylogenetic tree of the B_CAR_ dataset from Hispaniola was reconstructed under the best-fit nucleotide substitution model (GTR + I + G) using the MrBayes program (Ronquist et al., 2012). Two chains were run for 25 × 10^6^ generations, and stationarity (constant mean and variance of trace plots) and good mixing (effective sample size > 200) for all parameter estimates were assessed using TRACER v1.7 (Rambaut et al., 2018). The degree of phylogenetic mixing of B_CAR_ sequences obtained from both countries was then quantified using the BaTS program (Parker et al., 2008), which estimates phylogeny-trait associations using the Association Index (AI) (Wang et al., 2001), the Parsimony Score (PS) (Wang et al., 2001), and the maximum clade (MC) statistics. Results were considered significant for *p* < 0.01.

### Phylodynamic Analyses

The evolutionary rate, the age of the most recent common ancestor (*T*_MRCA_), the spatial diffusion pattern, and the rate of population growth (*r*, year^−1^) of B_CAR_ lineages in Hispaniola were jointly estimated using a Bayesian Markov Chain Monte Carlo (MCMC) approach implemented in BEAST v1.8 (Drummond et al., 2002; Drummond and Rambaut, 2007). Regression analyses using the TempEst program (Rambaut et al., 2016) revealed that the B_CAR_ *pol* dataset compiled here did not contain a sufficient temporal signal for reliable time-scale estimations [*X*-intercept (*T*_MRCA_) < 1910]. Thus, Bayesian MCMC analyses were performed using a relaxed uncorrelated lognormal molecular clock model (Drummond et al., 2006) with a uniform prior distribution on the substitution rate (1.7–3.0 × 10^−3^ subst./site/year), based on previous estimates (Hue et al., 2005; Zehender et al., 2010; Chen et al., 2011; Mendoza et al., 2014). Migration events were reconstructed using a reversible discrete phylogeographic model (Lemey et al., 2009) with a CTMC rate reference prior (Ferreira and Suchard, 2008). The number of location transitions (viral migrations between countries) throughout the evolutionary history was estimated using Markov jump counts (O’Brien et al., 2009). Changes in effective population size through time (*Ne*) were estimated using the non-parametric Bayesian skyline (BSKL) (Drummond et al., 2005) and Bayesian Skygrid (BSKG) (Gill et al., 2013) models. Estimates of the *r* were obtained under the best-fit parametric model selected using the log marginal likelihood estimation (MLE) based on the generalized stepping-stone sampling (GSS) method (Baele et al., 2016). Six MCMC chains were run for 200 million generations and then combined to ensure stationarity and good mixing as described above. The MC credibility (MCC) tree was summarized with TreeAnnotator v1.8 and visualized using the FigTree v1.4 program.

### Statistical Analyses

Gender and age group of Haitian individuals infected with different subtype B lineages were compared using Fisher’s exact test or *χ*^2^ implemented in Stata 13 software. Statistical significance was defined as *p* < 0.05.

## Results

**Figure 1:**
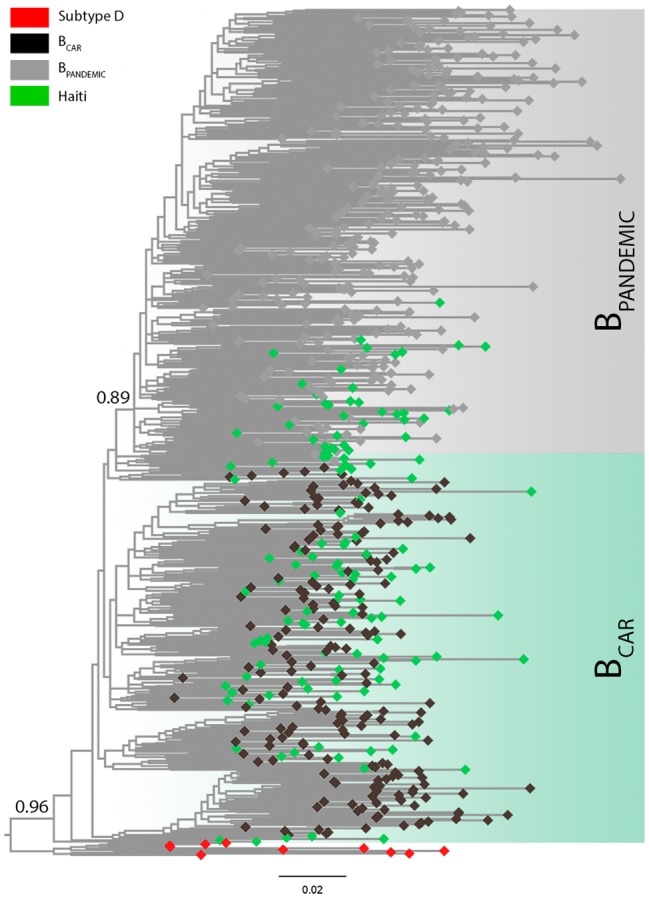
Classification of the HIV-1 subtype B sequences from Haiti among pandemic (B_PANDEMIC_) and non-pandemic (B_CAR_) lineages. ML phylogenetic tree of HIV-1 subtype B *pol* sequences (~1,000 nt) from Haiti (*n* = 127; green tips) together with representative sequences of the B_PANDEMIC_ (US = 165, France = 135; gray tips) and the B_CAR_ (Caribbean = 200; black tips) lineages. Node support (aLRT) for subtype B and B_PANDEMIC_ monophyletic groups are indicated. Shaded boxes highlight the position of the B_CAR_ and B_PANDEMIC_ lineages. Tree was rooted using HIV-1 subtype D reference sequences (red tips). The branch lengths are drawn to scale with the bar at the bottom indicating nucleotide substitutions per site.

All new HIV-1 *pol* sequences from Haiti obtained here (*n* = 127) were confirmed as non-recombinant subtype B by ML phylogenetic analysis (Supplementary Figure S1) and RDP recombination analysis (data not shown). The HIV-1 subtype B Haitian sequences were combined with viral strains representative of the B_CAR_ diversity in different Caribbean islands (*n* = 200) and of the B_PANDEMIC_ diversity in the US and France (*n* = 300) (Supplementary Table S2) as previously characterized (Cabello et al., 2014). The ML phylogenetic analysis revealed that most subtype B Haitian sequences (*n* = 91, 72%) were intermixed among basal non-pandemic B_CAR_ lineages, whereas the remaining ones (*n* = 36, 28%) branched within the well-supported (aLRT = 0.89) B_PANDEMIC_ clade (Figure 1). Analysis of the epidemiological characteristics of Haitian subjects showed that both B_CAR_ and B_PANDEMIC_ viral lineages circulated among males and females of different age groups (Supplementary Table S3). No significant differences were observed in the frequency of subtype B lineages according to gender (*p* = 0.82) or age group (*p* = 0.45), although subjects infected with B_CAR_ strains had a relative younger mean age (36.0 years) as compared with those infected with the B_PANDEMIC_ clade (40.1 years) (*p* = 0.03).

A closer inspection of the phylogenetic relationship among B_CAR_ sequences confirm that sequences from Haiti and the Dominican Republic were highly intermixed with each other and are usually basal to sequences from other Caribbean islands (Supplementary Figure S2). Most sequences from other well-sampled islands like Jamaica and Trinidad and Tobago, by contrast, branched in country-specific subclades that were nested within the Hispaniola B_CAR_ diversity. Very few sequences from Hispaniola (<1%) branched within Trinidadian and Jamaican clusters, confirming that most B_CAR_ infections in Hispaniola resulted from internal viral dissemination and not from re-introductions of viral strains from other Caribbean islands. To investigate the phylogeographic structure of the HIV-1 epidemic within Hispaniola, B_CAR_ *pol* sequences from Haiti here identified (*n* = 91) were combined with Haitian (*n* = 12) and Dominican (*n* = 123) B_CAR_ *pol* sequences identified in a previous study (Cabello et al., 2014) and analyzed using BaTS. Analyses of population subdivision rejected the null hypothesis of panmixis (i.e., complete intermixing of sequences from Haiti and the Dominican Republic) (Supplementary Table S4), demonstrating that despite frequent viral intermixing between both countries, the geographic subdivision of the HIV-1 B_CAR_ sequences from Hispaniola was greater than expected by chance.

**Figure 2:**
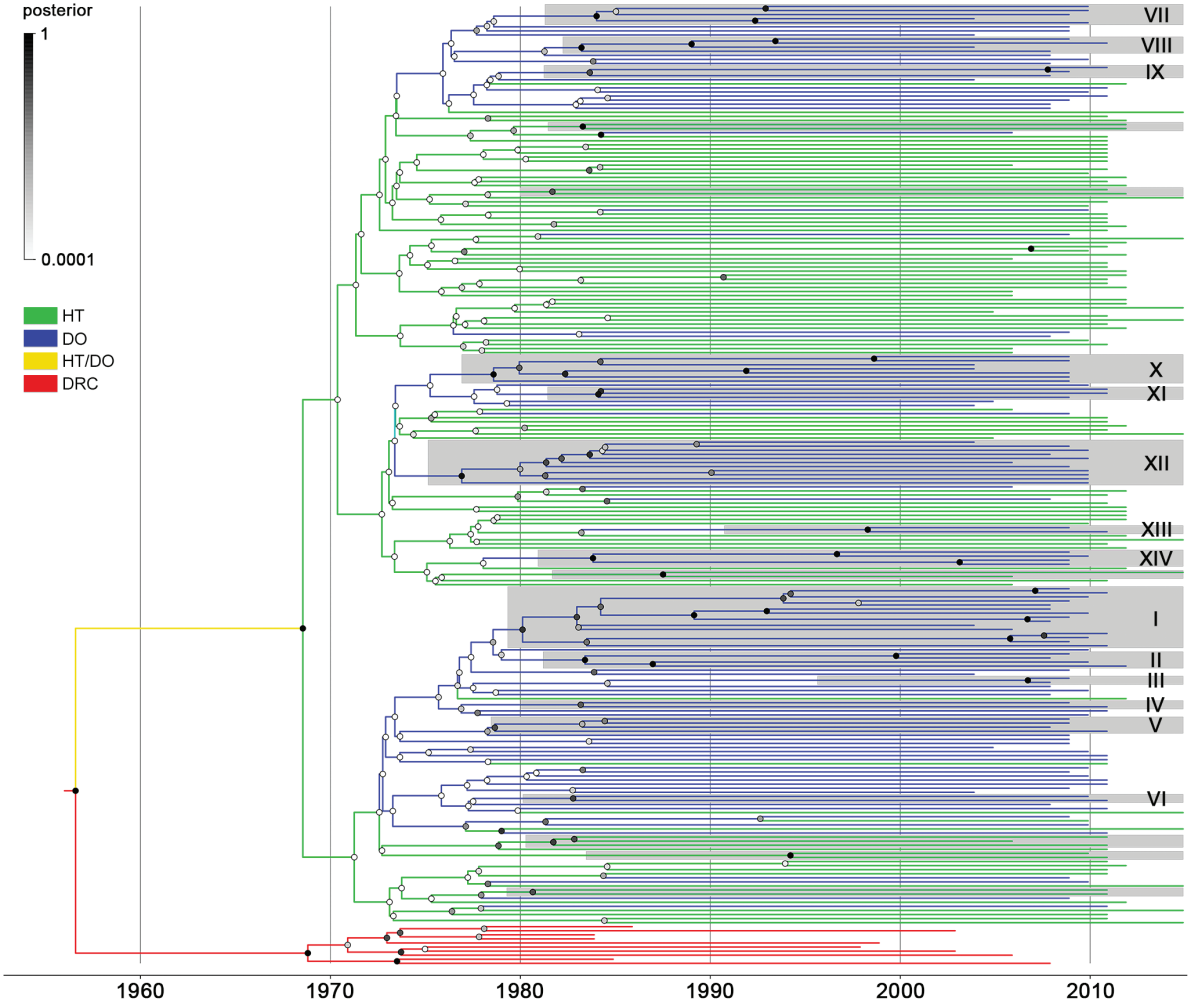
Spatiotemporal dissemination of HIV-1 B_CAR_ lineages in Hispaniola. Time-scaled Bayesian MCC tree of HIV-1 B_CAR_ *pol* sequences from Haiti (*n* = 103) and the Dominican Republic (*n* = 123) combined with subtype D reference sequences from the Democratic Republic of Congo (DRC; *n* = 10). Branches are colored according to the most probable location state of their descendent nodes as indicated in the legend on the left. Shaded boxes highlight the position of B_CAR_ clades only composed by sequences from Haiti or the Dominican Republic (identified by numbers) and that displayed both high clade (PP ≥ 0.70) and location state (PSP ≥ 0.90) node supports. Circles at internal nodes are colored according to the corresponding PP node support as indicated in the legend on the left. Branch lengths are depicted in units of time (years). The tree was rooted under the assumption of a relaxed molecular clock.

The same dataset of B_CAR_ *pol* sequences from Haiti (*n* = 103) and the Dominican Republic (*n* = 123) was then subjected to Bayesian phylogeographic analyses. Reconstruction of the spatiotemporal dissemination dynamic traced the T_MRCA_ for the HIV-1 B_CAR_ epidemic in Hispaniola at 1967 (95% HPD: 1961–1972), but failed to uncover its precise epicenter (Figure 2). After combining six independent Bayesian MCMC runs, the root location of the HIV-1 B_CAR_ ancestor in Hispaniola was traced with equal probability [posterior state probability (PSP) = 0.50] to Haiti and to the Dominican Republic. The difficulty to trace the location of the B_CAR_ root into a single country was also evidenced when the results obtained from independent Bayesian MCMC runs were visualized separately (Supplementary Table S5). Quantification of B_CAR_ flux between countries using Markov jump counts support a mean of 18 viral migrations from Haiti to the Dominican Republic and 9 viral migration events from the Dominican Republic to Haiti. These viral migrations started in the early 1970s and were homogenously distributed between the mid-1970s and the mid-2000s (Supplementary Figure S3). The Bayesian phylogeographic analysis also revealed 20 country-specific (14 Dominican and 6 Haitian) B_CAR_ monophyletic subclades with relative high node support [posterior probability (PP) > 0.70] (Figure 2). The Dominican B_CAR_ subclades mostly arose between the mid-1970s and the early 1980s (Supplementary Table S6) and together comprised 55% (*n* = 68) of B_CAR_ sequences from the country.

**Figure 3:**
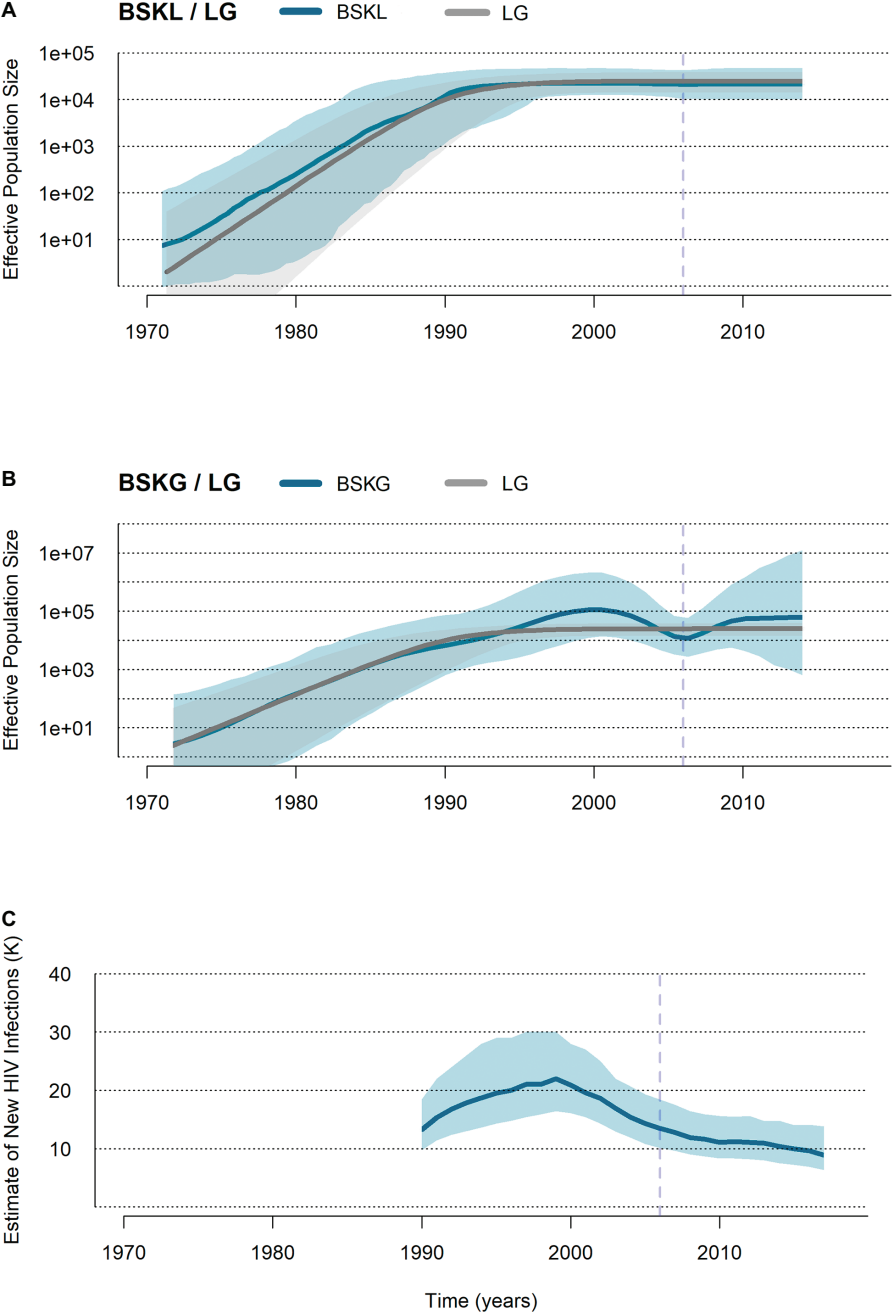
Population and epidemiological dynamics of the HIV-1 B_CAR_ epidemic in Hispaniola. **(A,B)** Plots showing the median (solid blue lines) and the 95% HPD intervals (dashed blue areas) estimates of the effective number of HIV-1 B_CAR_ infections (*Ne, y* axis) along time (years, *x* axis) in Hispaniola under the BSKL and BSKG models. The median *Ne* estimates provided by the logistic growth (LG) parametric model (dark gray line) and their 95% HPD (pale gray area) are co-plotted in both graphics. **(C)** Plot summarizing the number of new HIV cases in adult (>15 years old) populations from Haiti and the Dominican Republic according to the UNAIDS estimations (http://aidsinfo.unaids.org/). The dashed vertical lines indicate the time of the last coalescent event.

HIV-1 B_CAR_ sequences from Haiti (*n* = 103) and the Dominican Republic (*n* = 123) were finally used to reconstruct the demographic history of this viral epidemic in Hispaniola. Reconstruction of population dynamics with the BSKL coalescent-based model suggested that the B_CAR_ epidemic in Hispaniola experienced an initial phase of fast exponential growth until the beginning of the 1990s, followed by a stabilization of the *Ne* (Figure 3A). The BSKG model, however, supported a longer exponential growth phase until the late 1990s, followed by an epidemic decline that extended until the most recent coalescent event around the mid-2000s (Figure 3B). The UNAIDS epidemiological data, in agreement with the BSKG model, indicate a growth in the number of new HIV infections in Hispaniola until the early 2000s and a subsequent phase of decline extending until the most recent date (Figure 3C). To estimate the growth rate of the B_CAR_ epidemic at the initial phase, three parametric coalescent models (logistic, exponential, and expansion) were compared. According to the best-fit logistic growth coalescent model (Supplementary Table S7), the mean growth rate of the B_CAR_ epidemic during the first decades of expansion in Hispaniola was 0.50 (95% HPD: 0.37–0.65).

## Discussion

This study confirms that the HIV-1 subtype B epidemic in Haiti is mostly driven by dissemination of early divergent non-pandemic B_CAR_ lineages (Cabello et al., 2014). The relative prevalence of B_CAR_ lineages among HIV-1 subtype B infections in Haiti (72%) closely matches that estimated in the neighboring Dominican Republic (74%) (Cabello et al., 2014). Together, Haiti and the Dominican Republic host the largest population (~160,000 people) of B_CAR_-infected individuals in the Americas. Our results reveal a high degree of phylogenetic intermixing of the HIV-1 B_CAR_ sequences from Haiti and the Dominican Republic, consistent with the historical intense cross-border population mobility (Cohen, 2006; Rojas et al., 2011), but also support some level of geographic structure within Hispaniola. We estimate that nearly 55% of B_CAR_ infections in the Dominican Republic probably occurred within local transmission networks.

Previous evolutionary analyses (Gilbert et al., 2007; Junqueira et al., 2011; Worobey et al., 2016) support that the HIV-1 subtype B arrived in Haiti earlier than in any other American country. Here, we tested the hypothesis of the Haitian origin of subtype B epidemic by using for the first time a geographically balanced HIV-1 B_CAR_ dataset of Haitian (*n* = 103) and Dominican (*n* = 123) sequences. Our Bayesian phylogeographic analysis traced the root location of the B_CAR_ epidemic into Haiti and the Dominican Republic with the same posterior probability. This result revealed the complexity to uncover the geographic origin of the subtype B epidemic within Hispaniola by using only genetic data, probably due to the continuous cross-border viral movements between countries since the very early epidemic times. Thus, integration of non-genetic information (such as HIV incidence and prevalence data and human flows) into phylogeographic inference (Graf et al., 2015) would be probably indispensable to resolve the precise location of the HIV-1 B_CAR_ ancestor within Hispaniola.

Although phylogeographic analyses conducted here support the Haitian or Dominican origin of subtype B with the same probability, some epidemiological and historical data favor the Haitian origin hypothesis. The first AIDS cases were recognized in Haiti (1978–1979) a few years before those of the Dominican Republic (1983) and, by the early 1980s, HIV seroprevalence among Haitians was higher than among Dominicans (Pape et al., 1983; Koenig et al., 1987). Furthermore, the estimated T_MRCA_ of the B_CAR_ ancestor at around the late 1960s coincides with the return of Haitian professionals from the Democratic Republic of Congo (Gilbert et al., 2007), a country with an established HIV epidemic by that time (Worobey et al., 2008). By contrast, we found no similar historical link supporting relevant human flows between the Dominican Republic and the Democratic Republic of Congo during the 1960s.

Some epidemiological studies suggested that HIV-1 transmission between populations in Haiti and the Dominican Republic was uncommon during the early years and that tourists were the most likely source of first virus transmissions to Dominicans (Pape et al., 1983; Koenig et al., 1987). Our phylogeographic analysis, however, supports that HIV-1 B_CAR_ strains have been disseminated between Haiti and the Dominican Republic since the early 1970s and that several B_CAR_ transmission networks were already established in the Dominican Republic by mid-1970s, nearly a decade before the first AIDS cases were officially recognized in the country. The overall time scale here obtained for the B_CAR_ epidemic is fully consistent with that recovered in previous studies (Gilbert et al., 2007; Junqueira et al., 2011; Cabello et al., 2014; Worobey et al., 2016), supporting the reliability of our T_MRCA_ estimates. These results clearly indicate that HIV-1 B_CAR_ strains have been disseminated between Haitian and Dominican populations quite frequently since the early 1970s onwards.

While the BSKL model supports a stabilization of the B_CAR_ epidemic in Hispaniola from the early 1990s onwards, the BSKG reconstruction supports a sustained expansion until the late 1990s and a subsequent decline until the most recent coalescent event. The pattern here recovered by the BSKG model is consistent with the reported decline in HIV incidence in Haiti and the Dominican Republic since the late 1990s (UNAIDS, 2013, 2017), which is likely partially driven by changes in sexual behavior since the mid-1990s (Halperin et al., 2009). This finding is in agreement with previous studies that described that the BSKG model may uncover some aspects of the population history undetected by other Bayesian models (Gill et al., 2013; Mir et al., 2018). The mean growth rate estimated here for the B_CAR_ epidemic in Hispaniola during the first decades (0.50 year^−1^) is similar to those estimated for B_CAR_ and B_PANDEMIC_ lineages spreading in American countries with generalized heterosexual epidemics (0.35–0.45 year^−1^) (Cabello et al., 2014; Mendoza et al., 2014; Mir et al., 2015; Bello et al., 2018).

A drawback to consider in our study is the relative small size of our sample. According to the UNAIDS, the number of people living with HIV was estimated at around 150,000 in Haiti and 67,000 in the Dominican Republic in 2017. Assuming that 70–75% of those infections probably correspond to B_CAR_ viruses, a very small fraction (<1%) of B_CAR_-infected people living in those countries was included in our study. This low sampling density does not provide adequate power to assess HIV-1 clustering in generalized epidemics and could produce misleading results (Novitsky et al., 2014). The second limitation of our study is the lack of meta-data (such as city of origin, age, sex, or potential risk behavior) for most of the HIV-infected individuals included in our analysis, avoiding the identification of trends between individuals linked within the same local cluster. Finally, it is unclear whether our sample truly represents the whole diversity of HIV-1 B_CAR_ in Haiti because most Haitian individuals here analyzed were from the capital city (Port-au-Prince).

In summary, this study highlights that the HIV-1 epidemic in Haiti is mainly driven by dissemination of early divergent non-pandemic B_CAR_ strains. Our findings revealed that the HIV-1 B_CAR_ epidemics in Haiti and the Dominican Republic are highly connected by intensive bidirectional viral dispersal since the early 1970s and that local B_CAR_ transmission was already established in both countries when the first AIDS cases were officially recognized. Despite the use of a geographically balanced B_CAR_ dataset, probabilistic Bayesian phylogeographic models cannot uncover the root of the subtype B epidemic in Hispaniola by using only genetic sequence information. Our findings support that both national and bi-national coordinated prevention measures are necessary to further control the HIV-1 dissemination in Haiti and the Dominican Republic.

## Data Availability

All HIV-1 sequences were deposited in the GenBank database (accession numbers MK639799–MK639925).

## Ethics Statement

Comité National d’Ethique du Ministère de la Santé Publique et de la Population de Haiti (13/07/2009).

## Author Contributions

GS, GB, VL, and MN conceived and designed the study. GS, MO, JB, RC, and BL collected the samples and performed the HIV sequence amplification and genotyping. GB and IA performed the phylogenetic and phylodynamics inferences. MN performed the statistical analyses. GS, GB, IA, and VL wrote the manuscript. All authors analyzed the data and discussed and reviewed the manuscript.

### Conflict of Interest Statement

The authors declare that the research was conducted in the absence of any commercial or financial relationships that could be construed as a potential conflict of interest.
