# Supplementary material for: Phylogeographic Analyses Reveal the Early Expansion and Frequent Bidirectional Cross-Border Transmissions of Non-pandemic HIV-1 Subtype B Strains in Hispaniola
Source: Front Microbiol. 2019 Jun 26;10:1340. doi: 10.3389/fmicb.2019.01340 (PMC6622406; doi:10.3389/fmicb.2019.01340)
Supplement: Supplementary file 1 [file Data_Sheet_1.PDF]

Supplementary Material

Phylogeographic analyses reveal the early expansion and frequent bidirectional cross-border transmissions of non-pandemic HIV-1 subtype B strains in Hispaniola

Gonzalo Bello[1], Ighor Arantes[1], Vincent Lacoste[2], Marlene Ouka[3]; Jacques Boncy[4]; Raymond Césaire[3], Bernard Liautaud[5], Mathieu Nacher[6] and Georges Dos Santos[3]

***Correspondence:**

Georges Dos Santos

Georges.DOS-SANTOS@chu-martinique.fr

Supplementary Figures

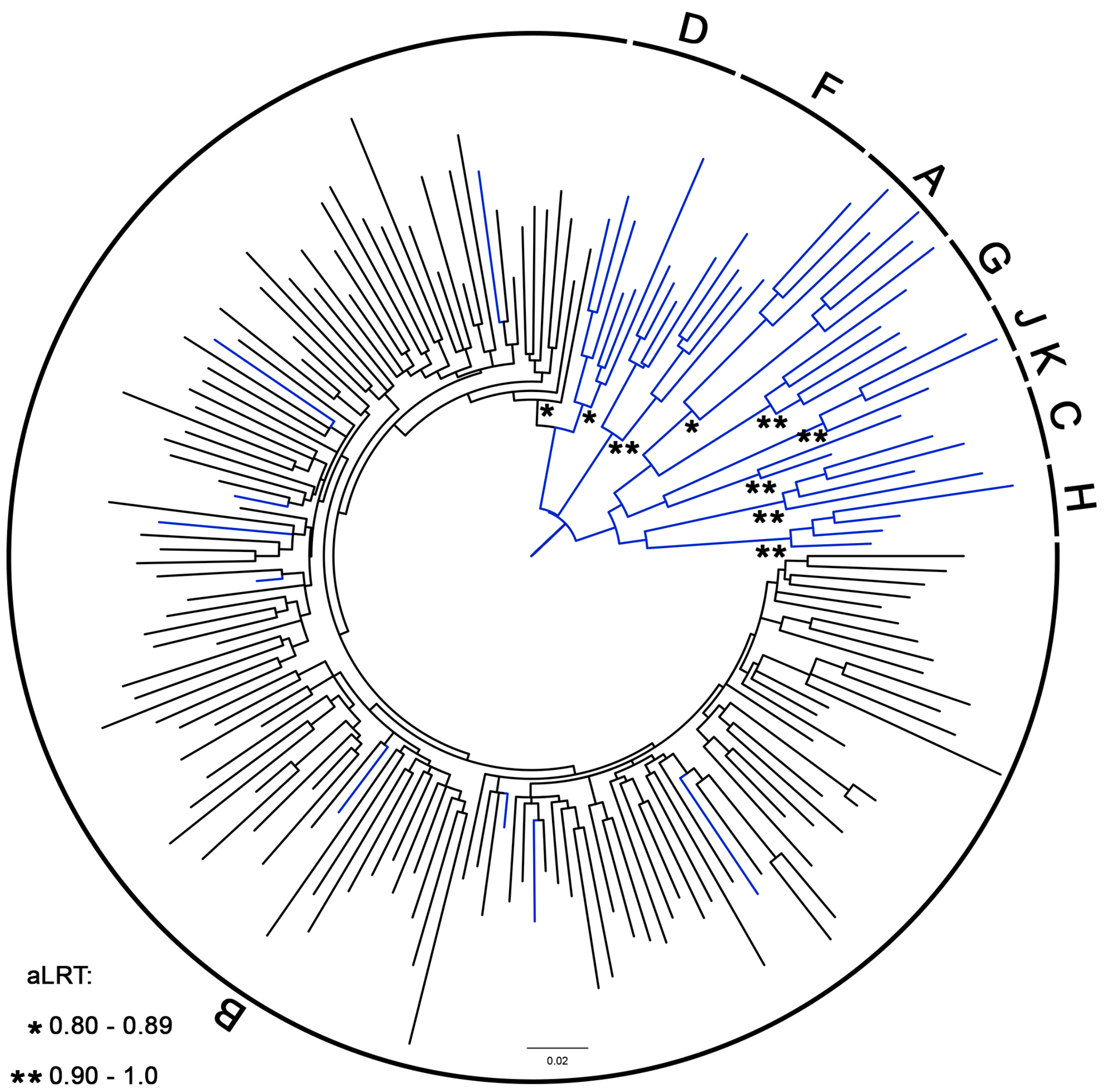

Supplementary Figure 1. ML phylogenetic tree of HIV-1 *pol* sequences from Haiti (black branches) and HIV-1 reference sequences of subtypes A-D, F-H, J and K (blue branches). The *a*LRT branch support value for each subtype is indicated. Tree was rooted at midpoint and the branch lengths are drawn to scale with the bar at the bottom indicating nucleotide substitutions per site.

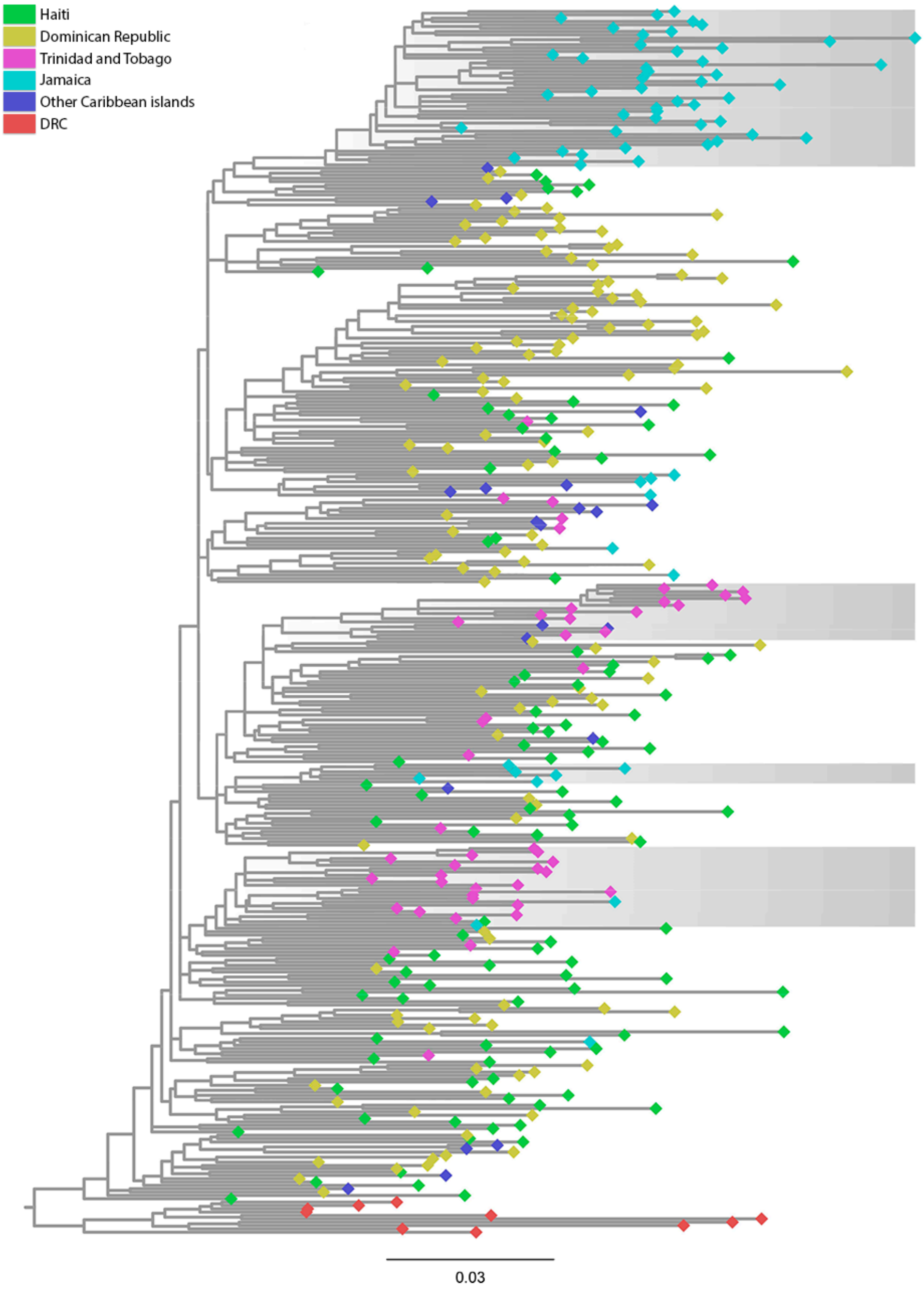

Supplementary Figure 2. ML phylogenetic tree of HIV-1 B_{CAR} *pol* sequences from Hispaniola and other Caribbean islands. Tips were colored according to the country of origin as indicate in the legend at top left. Shaded boxes highlight the position of the major country-specific B_{CAR} subclades detected in Jamaica and Trinidad and Tobago. Tree was rooted using HIV-1 subtype D reference sequences from DRC and the branch lengths are drawn to scale with the bar at the bottom indicating nucleotide substitutions per site.

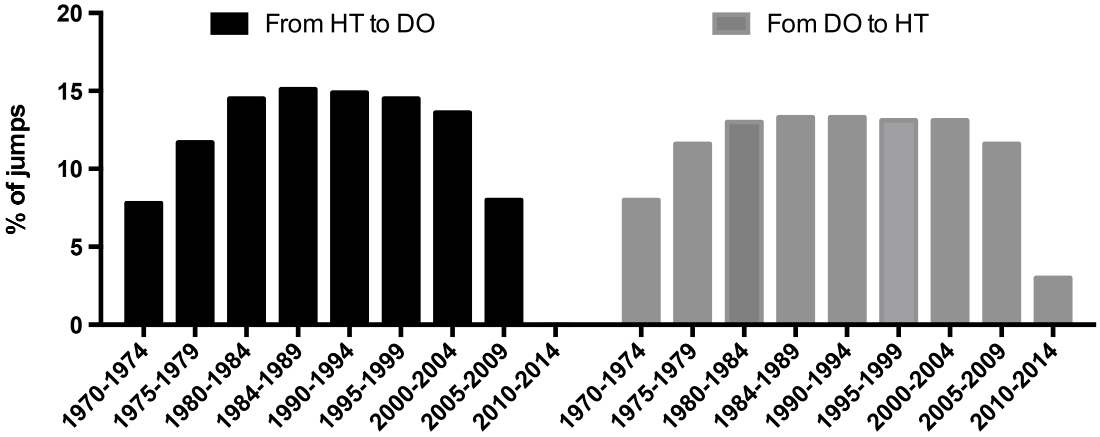

Supplementary Figure 3. Temporal distribution of Markov jump counts for the location transitions of the HIV-1 B_{CAR} strains between Haiti (HT) and the Dominican Republic (DO).

Supplementary Tables

Supplementary Table S1. HIV-1 subtype reference sequences used for subtyping.

Subtype	Clade	*N*	Accession numbers
A1/A2	-	3/3	AB253421 AB253429 AF286238 AF286237 DQ676872 GU201516
B	$B_{PANDEMIC}$	4	K03455 AY423387 AY173951 AY331295
	B_{CAR}	5	M17451 EU839601 EU839602 EU839603 EU839604
C	-	4	U52953 U46016 AF067155 AY772699
D	-	6	A07108 U88824 AF357631 AM041035 AY253311 AY371157
F1/F2	-	4/4	AF077336 AF005494 AF075703 AJ249238 AY371158 AJ249236 AJ249237 AF377956
G	-	4	U88826 AF061641 AF084936 AY612637
H	-	4	AF190127 AF190128 AF005496 FJ711703
J	-	3	AF082394 EF614151 GU237072
K	-	2	AJ249235 AJ249239

Supplementary Table S2. HIV-1 $B_{PANDEMIC}$ and B_{CAR} *pol* (PR/RT) reference sequences used for subtype B lineage assignment.

Clade	Country	*N*	Sampling time
$B_{PANDEMIC}$	France	135	1985-2008
	US	165	1997-2009
B_{CAR}	Dominican Republic	61	2005-2010
	Haiti	8	2004-2005
	Jamaica	62	2005-2010
	Trinidad and Tobago	48	2000-2003
	Other Caribbean countries [a]	21	2000-2004

[a] Antigua and Barbuda (n = 4), Bahamas (n = 5), Dominica (n = 1), Grenada (n = 2), Montserrat (n = 1), Saint Lucia (n = 4) and Saint Vincent and the Grenadines (n = 4).

Supplementary Table S3. Epidemiological information of subjects from Haiti infected by HIV-1 B_{CAR} and $B_{PANDEMIC}$ clades.

	Total B (n = 127)	B_{CAR} (n = 91)	$B_{PANDEMIC}$ (n = 36)
**Sex**			
Female	62 (49%)	46 (51%)	16 (44%)
Male	46 (36%)	32 (35%)	14 (39%)
Unknown	19 (15%)	13 (14%)	6 (17%)
**Age group (years)**			
18-24	9 (7%)	7 (8%)	2 (6%)
25-44	71 (56%)	54 (59%)	17 (47%)
45-66	28 (22%)	17 (19%)	11 (30%)
Unknown	19 (15%)	13 (14%)	6 (17%)

Supplementary Table S4. Statistical analysis of geographic structuring of HIV-1 B_{CAR} sequences from Haiti (HT) and the Dominican Republic (DO).

**Statistic**	**Observed value (95% CI)**	**Value under the null hypothesis (95% CI)**	***P*-value**
AI	7.52 (6.29-8.77)	12.42 (11.27-13.56)	0.0
PS	48.04 (44.0-52.0)	74.26 (70.22-77.79)	0.0
MC (HT)	6.10 (4.0-9.0)	4.02 (3.33-5.13)	0.03
MC (DO)	27.17 (19.0-36.0)	4.98 (4.11-6.23)	0.01

Supplementary Table S5. Root location of HIV-1 B_{CAR} sequences circulating in Haiti and the Dominican Republic.

**Run**	**Local**	**Posterior State Probability**
1	HT	0.58
	DO	0.42
2	HT	0.48
	DO	0.52
3	HT	0.48
	DO	0.52
4	HT	0.56
	DO	0.44
5	HT	0.37
	DO	0.63
6	HT	0.53
	DO	0.47
**Combined**	**HT**	**0.50**
	**DO**	**0.50**

Supplementary Table S6. Country-specific HIV-1 B_{CAR} transmission clusters detected in the Dominican Republic.

**Subclade**	***N***	**Posterior State Probability**	**Posterior Clade Probability**	**TMRCA (95% HPD)**
$B_{CAR-DO-I}$	15	1.0	0.82	1978 (1974-1983)
$B_{CAR-DO-II}$	4	1.0	0.97	1981 (1976-1987)
$B_{CAR-DO-III}$	2	1.0	1.0	2004 (2000-2007)
$B_{CAR-DO-IV}$	2	1.0	0.71	1981 (1975-1988)
$B_{CAR-DO-V}$	4	1.0	0.75	1978 (1974-1985)
$B_{CAR-DO-VI}$	2	0.95	0.78	1984 (1977-19991)
$B_{CAR-DO-VII}$	5	1.0	0.97	1981 (1976-1990)
$B_{CAR-DO-VIII}$	4	0.98	1.0	1983 (1977-1988)
$B_{CAR-DO-IX}$	3	0.99	0.74	1983 (1976-1990)
$B_{CAR-DO-X}$	7	0.99	0.97	1978 (1973-1983)
$B_{CAR-DO-XI}$	3	1.0	0.91	1981 (1975-1987)
$B_{CAR-DO-XII}$	11	1.0	0.92	1975 (1971-1980)
$B_{CAR-DO-XIII}$	2	0.98	1.0	1995 (1986-2002)
$B_{CAR-DO-XIV}$	4	0.98	1.0	1982 (1977-1988)

Supplementary Table S7. Best fit demographic model for the HIV-1 Bcar sequences from Haiti and the Dominican Republic.

**Clade**	**Model**	**GSS Log ml**	**Models compared**	**Log BF**
$B_{CAR–HISP}$	Log	-24738	-	-
	Expo	-24835	Log/Expo	97
	Expa	-24850	Log/Expa	112

Log marginal likelihood (ml) estimates for the logistic (Log), exponential (Expo) and expansion (Expa) growth demographic models obtained using the generalized stepping-stone sampling (GSS) method. The Log Bayes factor (BF) is the difference of the Log ml between alternative (H1) and null (H0) models (H1/H0). Log BFs > 3 indicates that model H1 is more strongly supported by the data than model H0.
